# Psychological health, wellbeing and COVID-19: Comparing previously infected and non-infected South African employees

**DOI:** 10.3389/fpsyg.2022.1013377

**Published:** 2022-11-03

**Authors:** Carin Hill

**Affiliations:** Department of Industrial Psychology and People Management, School of Management, College of Business and Economics, University of Johannesburg, Auckland Park, South Africa

**Keywords:** SARS-CoV-2, infected employees, mental health, burnout, DASS-21, psychological well-being

## Abstract

Most COVID-19 and work-related well-being research is centred around the adverse effects on employees’ psychological well-being and is not focused on the work-related well-being of those infected by SARS-CoV-2. Furthermore, COVID-19 and work-related well-being research is generally aimed at healthcare workers. The current study focused on investigating the difference in the level of burnout, anxiety, depression and stress between previously infected and uninfected participants. This study used a cross-sectional survey design and non-probability quota sampling to collect data. A retrospective pre-post design was used to determine the difference between the level of burnout of the participants before and after infection. Working adults in South Africa were targeted and divided into those previously infected (*n* = 245) and those not yet infected with COVID-19 (*n* = 221). Participants completed questionnaires relating to burnout, depression, anxiety, and stress. A comparison of means revealed a significant increase in burnout after being infected. Infected participants had significantly higher burnout, anxiety, depression, and stress levels than their non-infected counterparts. Emotional exhaustion, withdrawal, and stress were the most prevalent psychological ill-health problems. The results of this study indicated that a SARS-CoV-2 infection has a detrimental impact on participants’ psychological well-being and mental health compared to their own initially reported levels of burnout before infection, as well as compared to the levels of burnout, depression, anxiety and depression of the non-infected participants. Based on the findings, specific recommendations to industrial psychologists were made to manage the psychological impact of COVID-19 on employees.

## Introduction

Since 20 December 2019, the globe has experienced a ‘significant life event’ due to the Severe Acute Respiratory Syndrome Coronavirus 2 (SARS-Cov-2) and the subsequent COVID-19^1^ pandemic. The world underwent a critical change comparable to what people went through during World War II (Coleman, 2022), with profound economic, social, political, mental and physical health consequences (Douglas et al., 2020; Coleman, 2022). People across the globe have gone through stages of shock, unbelief, grief, bereavement and trauma, depression, sadness and fear (Berinato, 2020; Sahoo et al., 2020; Motamedzadeh et al., 2021; Pop-Jordanova, 2021). Everyday conversations focused on the uncertainty of the pandemic, when it would end, the effect of the pandemic on employment and the economy, and the possibility of contracting the virus. Several studies (Horesh and Brown, 2020; Qi et al., 2020; Salari et al., 2020; Bo et al., 2021; Janiri et al., 2021; Mohammadian Khonsari et al., 2021) showed that people worldwide experience the COVID-19 pandemic as a traumatic event and present with ensuing psychological symptoms, such as post-traumatic stress, depression, and anxiety, especially individuals recovering from COVID-19. Horesh and Brown (2020) reported that the COVID-19-induced stress-related mental health issues were highly correlated with characteristic elements of mass traumatic events.

Early in the pandemic, it became evident that employees were distressed and even traumatised by their experiences in the workplace. Workplaces had to reinvent how they operated and made decisions, confronting changing geographies, supporting staff virtually, and adapting to COVID-19 workplace regulations (Reuschke and Felstead, 2020; de Lucas Ancillo et al., 2021; Lee, 2021). Consequently, in the aftermath of COVID-19, employees had to learn new coping mechanisms that did not exist before the pandemic (Horesh and Brown, 2020) to deal with altered workplace practices and psychological stressors, such as fear of infection, job loss, social isolation and confinement (Hamouche, 2020; Kniffin et al., 2021).

More than 2 years into the pandemic, many organisations have embraced a ‘new normal’ way of work instead of anticipating a return to the status quo *after* the COVID-19 pandemic. Mckenzie et al. (2022) suggested that there “…may never be a ‘*post*-COVID world’, in the literal, posterior sense” (p: 1). Instead, the workplace has been forced to evolve due to redesign, social distancing, remote working, and security, so there will not be a return to the way the workplace used to operate (de Lucas Ancillo et al., 2021). Vyas (2022) expected the changes in the world of work to accelerate changes already being implemented pre-COVID-19, normalising ways of work previously thought of as unconventional or remodelling the pre-pandemic way of work. In this emerging ‘new normal’ way of work, with its inevitable changes, organisations must re-look and rethink policies and procedures from a different perspective to support staff members in the workplace (Greenwood and Anas, 2021). The consequence of these changes and ‘new normal’ work environment means that employees now have to adjust again to their work requirements and operationalisation after experiencing a traumatic event in their lives, some having been severely ill due to SARS-CoV-2 and some losing loved ones.

Greenwood and Anas (2021) found that mental health issues have seemingly become the norm in organisations; consequently, more people leave their jobs due to these issues. This supports the view of Sasaki et al. (2020) that vulnerable employees should be carefully supported during and after the pandemic to reduce employees’ psychological distress and maintain mental health and work performance. It is estimated that employees who recovered from being infected with SARS-CoV-2 need specific support, which warrants new support-programme designs and research (Vostanis and Bell, 2020). In general, all employees, those who were previously infected with SARS-CoV-2 and those who were not, need support as the long-term after-effect of COVID-19 needs to be identified effectively and managed accordingly (Horesh and Brown, 2020; Vostanis and Bell, 2020). To support employees optimally during the pandemic, new information is needed to inform managers on how to address the work-related well-being of employees affected by COVID-19 (Greenwood and Anas, 2021). With this in mind, to support staff functionally, it is essential to have information about the work-related wellbeing of employees who were infected with SARS-CoV-2 and to determine whether there are differences between previously infected and non-infected employees.

This study focuses on filling the knowledge gap pertaining to the psychological health and well-being experiences of previously infected *and* uninfected employees. Khawand and Zargar (2022) noted that there had been an upsurge in research endeavouring to understand the impact of COVID-19 on the psychological health of employees. However, not much is yet published on the psychological health and well-being of those infected by SARS-CoV-2, nor have there been many studies comparing work-related well-being between previously infected and uninfected participants. The studies focusing on infected patients’ psychological health and well-being (Badru et al., 2021; Mohammadian Khonsari et al., 2021) are primarily from the medical, health care and emergency professions (Giorgi et al., 2020). Giorgi et al. (2020) and Vostanis and Bell (2020) reviewed recent COVID-19 and well-being research and concluded that patients who suffered from SARS-CoV-2 and were hospitalised were more likely to present with anxiety, depression, and fatigue in the months that followed. Klaser et al. (2021) were among the few researchers who measured the prevalence of anxiety and depression symptoms in non-healthcare workers with and without prior SARS-CoV-2 infection. They found that the depressive and anxiety symptoms were related to a SARS-CoV-2 infection with a significant, albeit small, odds ratio, where infected participants had a 1.08 higher chance of experiencing these symptoms than those who had not been infected. Klaser et al. (2021) also noted that the relationship between depression and anxiety symptoms and having been infected with SARS-CoV-2 was the strongest for those participants who completed the questionnaire less than 30 days after being infected. Therefore, the current study aims to advance the knowledge base of employee psychological health and well-being within the context of COVID-19 by answering the following questions: (1) to what extent is there a difference in psychological health and well-being before and after being infected with SARS-CoC-2?; (2) is there a difference in psychological health and well-being experiences between previously infected and uninfected employees? and (3) which psychological health and well-being constructs are more prevalent among previously infected and uninfected employees?

To address these questions, the research reviewed psychological health and well-being literature, incorporating recent findings relating to the COVID-19 context, and explored the findings through the lens of the biopsychosocial model. The method section explains the research design, data collection, and measures used, followed by the data-analysis strategy, results, discussion of the findings and limitations of the study.

## Literature study

### Describing psychological health and mental well-being

Psychological health and mental well-being is a multi-faceted concept related to engaging activity, economic, emotional, mental, moral, physical, psychological, social, and spiritual functioning, as well as quality of life, life satisfaction and domain-specific satisfaction (Dodge et al., 2012; Mukhtar, 2020; Bergh, 2021). It should be noted that psychological health and well-being are correlated with but distinct from mental illness (Follmer and Jones, 2018). Gamm et al. (2003) differentiated between mental illness and mental health: Mental illness collectively represents all diagnosable mental disorders, when a person’s thoughts, behaviour, and mood are altered, causing some form of impairment and problems (for example, schizophrenia, affective disorders, anxiety disorders), while mental health “is a state of successful performance of mental function, resulting in productive activities, fulfilling relationships with other people, and the ability to adapt to change and to cope with adversity” (p: 97). According to Follmer and Jones (2018), psychological health and well-being (or the lack thereof) are a work outcome where the affective state is generally momentary and a normal response to specific circumstances.

Psychological health and well-being can be depicted as a continuum that is evident in the bipolarity of its psychological constructs (Johnson and Wood, 2017): happiness vs. depression, calmness vs. anxiety, distress vs. eustress, vigour vs. fatigue, optimism vs. cynicism, dedication vs. apathy, and cognitive weariness vs. cognitive absorption (González-Romá et al., 2006; Rothmann, 2008; Asiwe et al., 2014; Johnson and Wood, 2017). In general, psychological health and mental well-being represent the absence of affective states such as depression, anxiety, fear, and stress (Bergh, 2021) and syndromes such as burnout (Fraga, 2019). Thus, a low state of well-being can presumably indicate the presence of psychological ill-health that includes depression, anxiety, stress, and burnout, while higher states of well-being may reflect engagement, eustress, happiness, and flourishing (Hall et al., 2016; Johnson and Wood, 2017; Querstret et al., 2020). The current article focuses on burnout, depression, anxiety, and stress.

#### Burnout

Burnout differs from stress since it often involves prolonged exposure to work-related stress, causing burnout (Schaufeli, 2021). Experiencing chronic fatigue because of high job demands, detachment from work and colleagues, increased cynicism, and a sense that the person does not accomplish as much in their work as usual (Maslach, 1996; Maslach et al., 2001) all characterise a state of burnout. Studies show that burnout symptoms often appear similar to symptoms of depression, such as loss of concentration, exhaustion and fatigue (Schaufeli and Enzmann, 1998; Koutsimani et al., 2019). However, depression can develop irrespective of a person’s environment or psychosocial situation, while the onset of burnout is specifically related to a person’s work environment (Koutsimani et al., 2019).

#### Depression

According to Lovibond and Lovibond (1995), depression is “characterised principally by a loss of self-esteem and incentive, and is associated with a low perceived probability of attaining life goals of significance for the individual as a person” (p: 342). Causes of depression are generally clustered under adverse life events on the one hand, and factors related to achievement, characterological, childhood, existential, hormonal, interpersonal conflict, intimacy, neglect, physiological, and relationships on the other (Addis et al., 1995; Piccinelli and Wilkinson, 2000; Beurel et al., 2020). Kessler and Bromet (2013) review of the literature summarises the burden of disease for depression as:

Difficulties in role transitions (e.g., low education, high teen childbearing, marital disruption, unstable employment), reduced role functioning (e.g., low marital quality, low work performance, low earnings), elevated risk of onset, persistence and severity of a wide range of secondary disorders, and increased risk of early mortality due to physical disorders and suicide (p. 119).

Certain somatic consequences include the risk of cardiovascular diseases, diabetes, stroke, obesity (Wulsin et al., 1999; Penninx et al., 2013) and sleep disturbances (Fang et al., 2019).

#### Anxiety

Anxiety represents an emotional state that can change over time and in intensity and represents a personality trait that differentiates how dangerous and threatening people perceive the world around them (Spielberger, 1966, 1972). Anxiety can be described as a complex reaction to a perceived threatening situation that includes feelings of tension, apprehension, fear, and worry (emotional reaction), intensified arousal of the autonomic nervous system and skeletal muscle effects (physiological reaction), as well as fretting and experiencing unpleasant thoughts and worries (cognitive reaction; Spielberger, 1972; Clark and Watson, 1991; Lovibond and Lovibond, 1995; Psychountaki et al., 2003).

#### Stress

Stress is defined as a physical and psychological response of the body to any demand that threatens a person’s physical and mental well-being (Sharma, 2018; Bergh, 2021). According to Peters et al. (2021), stress is generally regarded as pathogenic and detrimental to a person’s immune system. Stress causes physical and psychological ill-health, including symptoms such as a weakened immune system (Peters et al., 2021), effects on the digestive system (Sharma, 2018), cardiovascular disease and diabetes (Sharma, 2018; Seiler et al., 2020), colds and flu (Seiler et al., 2020; Peters et al., 2021), problems in sleeping, emotional problems, depression, anxiety and panic attacks (Devi et al., 2019). Moreover, Seiler et al. (2020) report that the vulnerability to certain types of cancer could be increased by the influence of chronic stress on protective immune responses in the body.

### Psychological health and well-being during COVID-19

According to Qiu et al. (2020), adverse psychological outcomes such as depression, anxiety, and panic disorder were triggered or exasperated during the pandemic. The COVID-19 pandemic in general, regardless of infection status, adversely affects individuals’ psychological health, resulting in the prevalence of stress, depression, burnout, trauma, post-traumatic stress, and anxiety (Qiu et al., 2020; Restauri and Sheridan, 2020; Yıldırım and Solmaz, 2020). Examples of these studies include that of Campbell and Gavett (2021), who reported mental health declines during the pandemic, indicating the prevalence of burnout, anxiety, feeling exhausted and isolated, increased job demands and growing disengagement at work. Pretorius and Padmanabhanunni (2021) found in their study among young adults in South Africa that exceptionally high levels of anxiety, loneliness, and decreased life satisfaction were present during the COVID-19 pandemic. In a 2021 study of mental health, 76% of full-time employees in the United States reported that during the past year they experienced an effect on their mental health in the form of symptoms of burnout, depression, and anxiety (Mind Share Partners’ Mental Health at Work, 2021). However, these studies focused on psychological health and well-being within the general context of the pandemic and did not take infection status into account.

Several international studies indicate that SARS-CoV-2 survivors show a risk of developing post-traumatic stress disorder (Qiu et al., 2020; Stamu-O’Brien et al., 2020; Janiri et al., 2021; Sekowski et al., 2021; Tarsitani et al., 2021), fatigue, anxiety and depression (Qiu et al., 2020; Sahoo et al., 2020). Mohammadian Khonsari et al. (2021) described how health care professionals in Iran who had been infected with SARS-CoV-2 showed a high risk of displaying psychological symptoms such as depression, stress and anxiety. Similarly, other studies (Qi et al., 2020; Sahoo et al., 2020; Zhang et al., 2020) found an increased prevalence of depression and psychological morbidity among patients hospitalised for SARS-CoV-2. Qi et al. (2020) reported on their study among recovering patients that their mental health problems were alarming. The patients reported that COVID-19 was not merely an illness for these individuals but rather a “life-changing disastrous experience which not only impairs physical well-being but also their mental health” (Qi et al., 2020, p: 9). Of these studies, only Mohammadian Khonsari et al. (2021) included non-infected participants in their samples. Kim et al. (2021) found that 20% of uninfected South Koreans reported high levels of depression during the COVID-19 pandemic. However, Rehman et al. (2022) established that among the uninfected Indian population, mean values of depression, anxiety and stress decreased significantly despite an increase in confirmed SARS-CoV-2 cases. These two studies, however, focused only on uninfected participants.

Only a few studies compared the psychological health and well-being of infected and non-infected participants (Risal et al., 2020; Shi et al., 2020; Wang et al., 2020; Zhang et al., 2020; Mohammadian Khonsari et al., 2021; İkiışık et al., 2022) and the results are diverse. According to Shi et al. (2020), compared to the non-infected, Chinese individuals previously infected with SARS-CoV-2 were twice as likely to develop mental-health symptoms such as depression, anxiety, insomnia, and acute stress. Healthcare professionals infected with SARS-CoV-2 also showed a higher risk of displaying psychological stress than their colleagues who were not infected (Mohammadian Khonsari et al., 2021). Depression was significantly more prevalent among healthcare workers (Mohammadian Khonsari et al., 2021), medical students (Risal et al., 2020) and the general Chinese public (Zhang et al., 2020) who were positive for COVID-19, while no significant differences in depression were found between infected and uninfected patients with multiple sclerosis (Broche-Pérez et al., 2021) or among Turkish municipal workers (İkiışık et al., 2022). Anxiety was significantly more frequent among patients with multiple sclerosis (Broche-Pérez et al., 2021), medical students (Risal et al., 2020), and healthcare workers (Mohammadian Khonsari et al., 2021) who experienced SARS-CoV-2 infection. In contrast, no significant differences in anxiety were found between infected and uninfected participants from the general Chinese public (Zhang et al., 2020) or Turkish municipal workers (İkiışık et al., 2022). Though these studies compared the infected and uninfected, most of these studies focused only on depression and anxiety and none included burnout. Furthermore, no studies have been found that compare the level of psychological health and well-being of the infected and non-infected within the South African context or that compare pre-and post-infection burnout levels.

### Theoretical setting

In Engel (1977), introduced the biopsychosocial model as an alternative approach to how physicians can deal with patients and their problems by taking into account physiologic, psychological, and social factors. The biopsychosocial model incorporates the disease, an objective biological event that damages individuals physiologically, and the illness, which refers to the psychological and social response of the sick individuals and their relations to the sickness (Turk and Monarch, 2002; Gatchel and Kishino, 2012). Although the model is generally applied within the medical field, it can be operationalised within the context of COVID-19. Jadoo (2020) explained the biopsychosocial model within the context of the COVID-19 pandemic as follows: the *bio* entity relates to the actual viral infection and the physiological effect it has on a person’s body; the *psycho* entity relates to the thoughts and behavioural and emotional reactions to being infected and to the sudden changes in lifestyle; and the *social* entity incorporates social aspects such as isolation and a lack of interaction with others that can influence a person’s recovery and rehabilitation. According to Wainwright and Low (2020), the rehabilitation process of someone recovering from SARS-CoV-2 requires a holistic approach that takes the person and their physical, psychological and social factors and supportive needs into account. Therefore, the biopsychosocial model offers a comprehensive framework to aid in understanding the interplay between psychological health and well-being and being infected with SARS-CoV-2.

## Materials and methods

Ethical clearance was obtained from an institutional review board (IPPM-2021-567) and, in accordance with the clearance granted, the study adhered to all ethical requirements.

### Data collection

To determine the sample size to compare pre-and post-scores where an effect size of Cohen’s *d_z_* = 0.50 with an 80% power (alpha = 0.05, two-tailed) would be ensured, G*Power (Faul et al., 2007, 2009; Bartlett, 2019) indicated a sample size of 34 participants was needed for a paired-samples *t*-test. For an effect size of Cohen’s *d_z_* = 0.50 with a 95% power (alpha = 0.05, two-tailed), G*Power suggested a sample size of 54 participants was needed. To compare the infected and uninfected participants while ensuring an effect size of Cohen’s *d* = 0.50 with 80% (alpha = 0.05, two-tailed), G*Power recommended that a sample of 64 participants per group (*N* = 128) would be needed to conduct an independent samples *t*-test. For an effect size of Cohen’s *d_z_* = 0.50 with a 95% power (alpha = 0.05, two-tailed), G*Power suggested a sample size of 105 participants per group (*N* = 210) was needed.

This study used a cross-sectional survey design and non-probability quota sampling to collect data. Participants were screened based on whether they had been infected with SARS-CoV-2. A retrospective pre-post design was used to determine the difference between the level of burnout of the participants’ pre and post being infected with SARS-CoV-2. The retrospective pre-post design allows the researchers to collect retrospective pre-test and current (post-test) data at the same time (Little et al., 2020) and is “ideal for assessing variables related to events, such as the COVID-19 pandemic, that occur without notice or intent” (p: 4; Miller et al., 2020). According to Drennan and Hyde (2008), using a retrospective pre-post design reduces response shift bias – a person’s overestimated evaluation of the impact or level of a certain construct. Participants who reported that they had been infected previously with SARS-CoV-2 were invited to complete an additional section in the questionnaire where they had to evaluate their level of burnout prior to being infected with SARS-CoV-2 in addition to answering questions relating to their current level of burnout. A total of 500 participants completed the questionnaire; however, after removing multivariate outliers, the original dataset was reduced to 466.

QuestionPro was used to collect data. The researchers obtained participants through their personal networks and working South African adults on the Prolific platform.^2^ The study was presented on the Prolific platform as a study on ‘The work-related well-being of employees who have/have not had COVID-19’, and participants were financially rewarded for completing the short survey. Participants had to be 18 years old and be an employee in South Africa for at least 1 year. The participants consented to complete the survey, understanding that their participation in this study was voluntary and that they could discontinue or withdraw without any adverse consequences. The confidentiality of the questionnaire and research process was explained and the electronic datasets were anonymised and stored on a password-protected computer. The participants were informed that only the researchers involved in the project would have access to the data. Since some of the items could seem potentially sensitive (for example, questions regarding COVID-19 status), the participants were notified that all the answers would be kept anonymous and they could therefore feel free to answer honestly. Lastly, for those participants who may have needed immediate support for any suspected mental health condition or COVID-19 related help, the local contact numbers for the South African Depression and Anxiety Group and the South African COVID-19 Hotline were provided at the beginning and at the end of the survey.

### Sample

For the current study, data were collected from employed South African participants (*N* = 466) who had been infected with SARS-CoV-2 (*n* = 245) and those who had not been infected with SARS-CoV-2 (*n* = 221). Table 1 shows that most of the participants (*n* = 23.4%) who had previously been infected with SARS-CoV-2 experienced a moderate level of infection severity. Participants were primarily female (66%). The participants’ ages ranged from 18 to 68 years (M = 32.63; SD = 7.72), mainly employed full-time (89%). The intermediate work positions (34.3%), as well as first-level management (19.5%), and middle management (27.5%) positions were mostly represented by the sample. Lastly, the respondents worked mostly in for-profit industries (52%). However, non-profit organisations (5%), government entities (15%), and the educational (11%) and healthcare (5%) sectors were also represented.

**Table 1:** Demographic characteristics of the sample.

**Characteristic**	**Category**	**Frequency**	**Percentage**
Previous SARS-CoV-2 infection	Yes	245	52.6%
	No	221	47.4%
If yes, how severe was the infection?	Mild	72	15.50%
	Moderate	109	23.40%
	Severe	59	12.70%
	Critically ill / hospitalized	4	0.90%
	Other	1	0.20%
Sex	Male	157	33.7%
	Female	309	66.3%
Age	<31	217	46.6%
	31–40	185	39.7%
	>40	60	12.9%
	*Missing*	*4*	*0.9%*
Type of employment	Fulltime	416	89.3%
	Part-time	44	9.4%
	*Missing*	*6*	*1.3%*
Work-level	Entry level	39	8.4%
	Intermediate level	160	34.3%
	First-level management	91	19.5%
	Middle management	128	27.5%
	Senior management	29	6.2%
	Top-level management	8	1.7%
	Owner	11	2.4%
Industry	For-profit	241	51.7%
	Non-profit	22	4.7%
	Government	69	14.8%
	Education	49	10.5%
	Healthcare	21	4.5%
	Other	64	13.7%

### Measures

All the measures used in the current study were open-access instruments.

#### The South African burnout scale

The South African Burnout Scale (SABOS) was developed by Asiwe et al. (2014) to measure cognitive weariness (six items; ‘not being able to concentrate while at work’), emotional exhaustion and withdrawal (six items; ‘feeling less connected to my work’), and fatigue (five items; ‘not having enough energy to go to work in the morning’) aspects of burnout among working individuals. Participants were asked to respond to the 17 statements that described ways they may feel about their work responsibilities and work environment and rate their experiences on a seven-point frequency scale ranging from ‘never’ to ‘always’. Asiwe et al. (2014) reported high internal consistency for the three scales, ranging between 0.82 and 0.88. The Cronbach’s alpha coefficients for the current study were 0.96 for Cognitive Weariness, 0.93 for Fatigue, and 0.92 for Emotional Exhaustion/Withdrawal. The model fit of the SABOS was acceptable after allowing the correlation of certain error co-variances [*χ^2^* = 474.87 (*n* = 466), *χ^2^/df* = 4.24, RMSEA = 0.08, TLI = 0.95, CFI = 0.96].

Participants who had previously been infected with COVID-19 were asked to provide two sets of ratings simultaneously: the first was a retrospective rating on how they felt about their work responsibilities and work environment prior to being infected with COVID-19, and the second related to how they felt about their work responsibilities and work environment at the time of survey completion (i.e., after being infected with COVID-19). The retrospective rating’s internal consistency for this sample was 0.91 for Cognitive Weariness, 0.80 for Fatigue, and 0.87 for Emotional Exhaustion/Withdrawal. After allowing certain error co-variances to correlate, the model fit for the retrospective SABOS proved to be acceptable [*χ^2^* = 239.37 (*n* = 245), *χ^2^/df* = 2.18, RMSEA = 0.07, TLI = 0.94, CFI = 0.95].

#### The depression, anxiety and stress scale

The 21-item version of the Depression Anxiety and Stress Scales (DASS-21) was developed by Lovibond and Lovibond (1995) and includes three self-report scales intended to determine a person’s emotional states in terms of depression (seven statements), anxiety (seven statements), and stress (seven statements). According to Lovibond and Lovibond (1995), the DASS-21 measures depression symptoms, including inertia, lack of interest/involvement, dysphoria, hopelessness, self-deprecation, and inability to enjoy normal activities and to appreciate life (for example, ‘I felt down-hearted and blue’). The anxiety scale measures a person’s autonomic arousal, skeletal muscle effects, situational anxiety, and subjective experience of anxious affect (for example, ‘I was aware of dryness of my mouth’; Lovibond and Lovibond, 1995). Lastly, the stress scale measures the extent to which a person has difficulty relaxing, nervous arousal, and how easily upset, agitated, irritated and impatient a person gets (for example, ‘I found it difficult to relax’). Participants had to indicate how much the statement applied to them over the past week on a four-point rating scale ranging from ‘Did not apply to me at all’ to ‘Applied to me very much or most of the time’. Lee (2019) found internal consistency coefficients of 0.90 for the Depression scale, 0.82 for the Anxiety scale, and 0.87 for the Stress scale. The reliability coefficients for the current study were excellent (Depression α = 0.91; Anxiety α = 0.87; Stress α = 0.89). The initial model fit was acceptable [*χ^2^* = 239.37 (*n* = 245), *χ^2^/df* = 2.18, RMSEA = 0.07, TLI = 0.94, CFI = 0.95], but after allowing certain co-variances to correlate, the model fit for the DASS-21 was good [*χ^2^* = 528.03 (*n* = 466), *χ^2^/df* = 2.97, RMSEA = 0.07, TLI = 0.94, CFI = 0.95].

### Data analysis

Data were cleaned and analysed using SPSS 27.0. Paired-samples *t*-tests and independent-samples *t*-tests were used to analyse the data. Statistical significance was set at *p* < 0.05 (two-tailed), and the exact *p*-values were given for all analyses. Levene’s Test for Equality of Variances was inspected to determine whether equal variances could be assumed. If equal variances could not be assumed for the factor, the results for ‘equal variances not assumed’ were reported.

A paired-samples *t*-test was used to compare the level of participants’ burnout prior to and after being infected with SARS-CoV-2. For the current study, 245 cases were subjected to the paired-samples t-test, and this sample size was sensitive to the effects of Cohen’s *d* = 0.18 with 80% power or 0.23 with 95% power (alpha = 0.5, two-tailed). Therefore, the study would not reliably be able to identify effects smaller than Cohen’s *d* of 0.18. Cohen’s *d* uses the sample’s standard deviation of the mean difference to indicate the effect size of the results.

Independent *t*-tests were applied to measure the differences in burnout, depression, anxiety and stress between participants who had previously been infected and those who had not been infected with COVID-19. In this study, the sample sizes adhered to this recommendation, since there were 245 participants for the group that had been previously infected with SARS-CoV-2 (group 1), while there were 221 participants for the group that had not been infected with SARS-CoV-2 at the time of the study (group 2). A sensitivity power analysis (G*Power; Faul et al., 2007, 2009) showed that an independent samples *t*-test with 245 participants for group 1 and 221 participants for group 2 (*N* = 466) would be sensitive to the effects of Cohen’s *d* = 0.26 at 80% power or 0.34 at 95% power (alpha = 0.05, two-tailed). Therefore, the study would not reliably be able to detect effects smaller than Cohen’s *d* = 0.26.

## Results

### Mean differences in burnout scores pre-and post-SARS-CoV-2 infection

**Figure 1:**
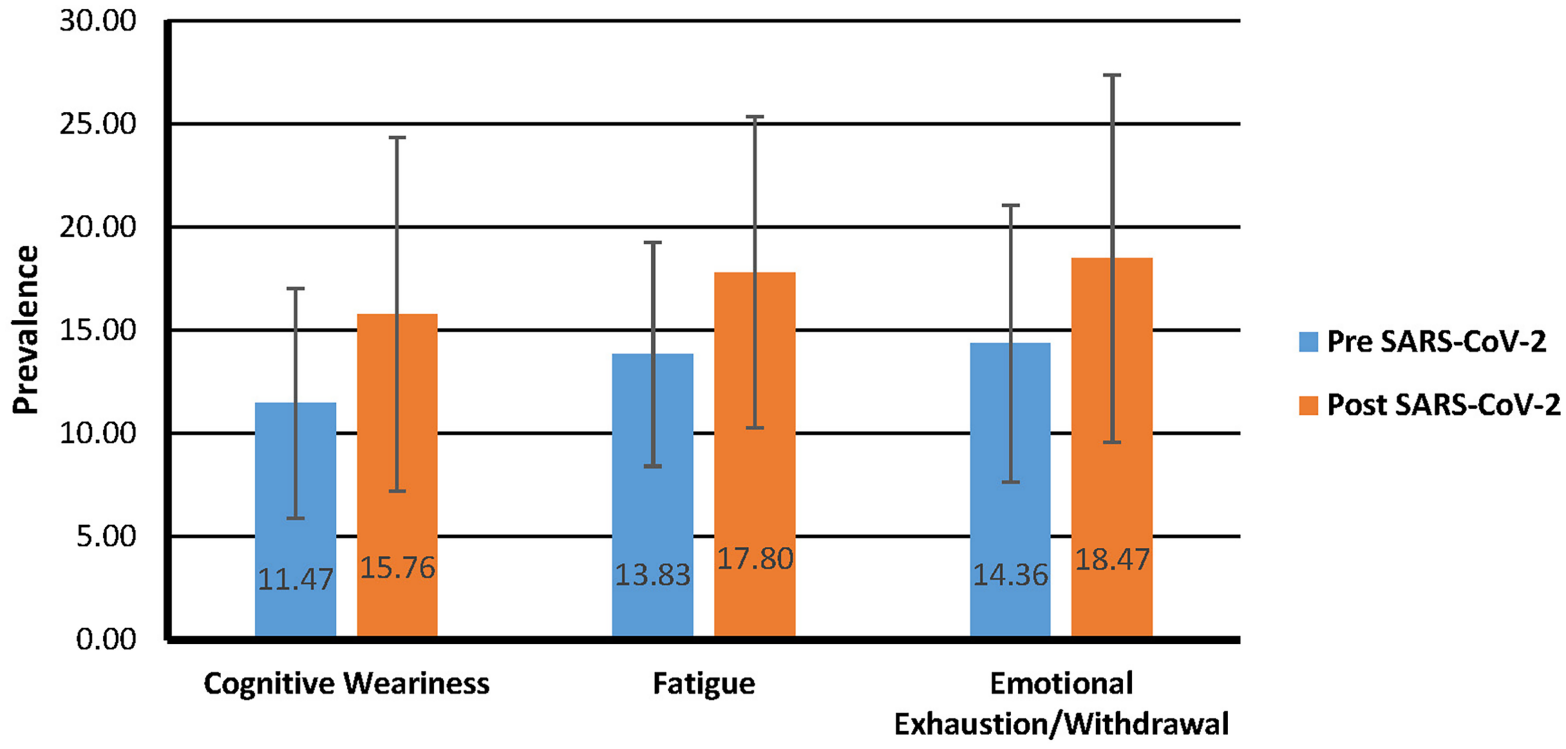
Pre vs post SARS-CoV-2 infection comparison of burnout mean scores.

A paired-samples *t*-test was performed to assess the difference in the level of burnout of participants before and after having been infected with SARS-CoV-2. The pre-and post-means for the three burnout scales are presented in Figure 1. There were statistically significant increases for all three burnout scales before being infected to after being infected: Cognitive Weariness [*M* increase = 4.29, *SD* increase = 7.71, CI = 3.32–5.26, *t*(244) = 8.71, *p* = 0.000], Fatigue [*M* increase = 3.96, *SD* increase = 7.23, CI = 3.05–4.87, *t*(244) = 8.58, *p* = 0.000], and Emotional Exhaustion/Withdrawal [*M* increase = 4.11, *SD* increase = 6.55, CI = 3.29–4.93, *t*(244) = 9.82, *p* = 0.000]. Cohen’s *d* indicated the increases were moderate too large for all three scales (Cognitive Weariness = 0.56; Fatigue = 0.55, Emotional Exhaustion/Withdrawal = 0.63) and indicative that the test was sufficiently sensitive to the minimum effect size at 95% power.

### Psychological well-being differences between previously infected and non-infected groups

#### Burnout

**Figure 2:**
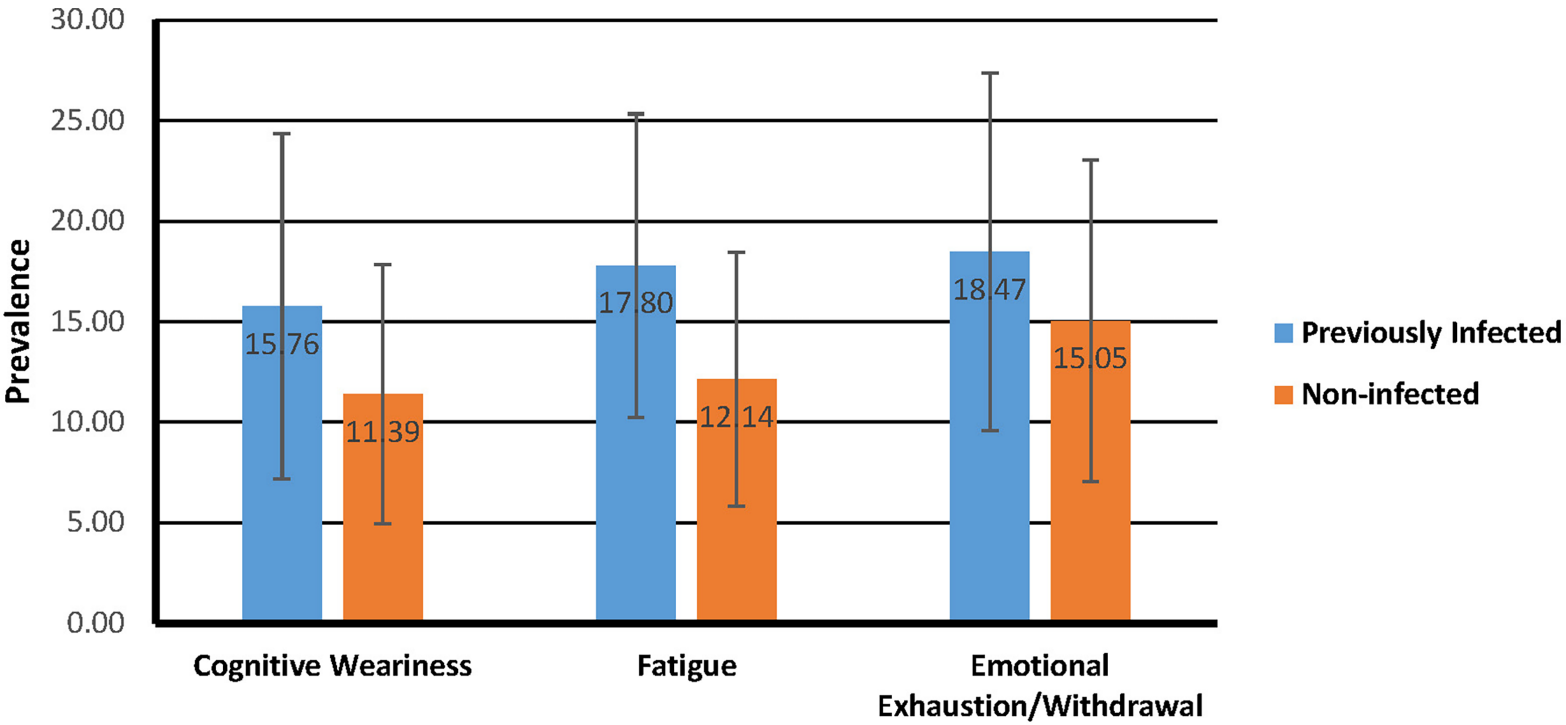
Infected vs non-infected comparison of burnout mean scores.

An independent-samples *t*-test was executed to measure the difference in the level of burnout among those who have been infected with SARS-CoV-2 and those who have not yet been infected with SARS-CoV-2 (Figure 2). The overall results indicated significant differences between the two groups for all three burnout scales: Cognitive Weariness [*M* difference = 4.37, *SD* difference = 0.70, CI = 2.99–5.74, *t*(449.63) = 6.25, *p* = 0.000], Fatigue [*M* difference = 5.66, *SD* difference = 0.64, CI = 4.39–6.92, *t*(461.36) = 8.81, *p* = 0.000], and Emotional Exhaustion/Withdrawal [*M* difference = 3.42, *SD* difference = 0.79, CI = 1.88–4.97, *t*(464) = 4.35, *p* = 0.000]. Cohen’s *d* indicated the increases were moderate too large for the different scales (Cognitive Weariness = 0.57; Fatigue = 0.81, Emotional Exhaustion/Withdrawal = 0.40). The test was therefore sufficiently sensitive based on the minimum effect size at 95% power.

#### Depression, anxiety and stress

**Figure 3:**
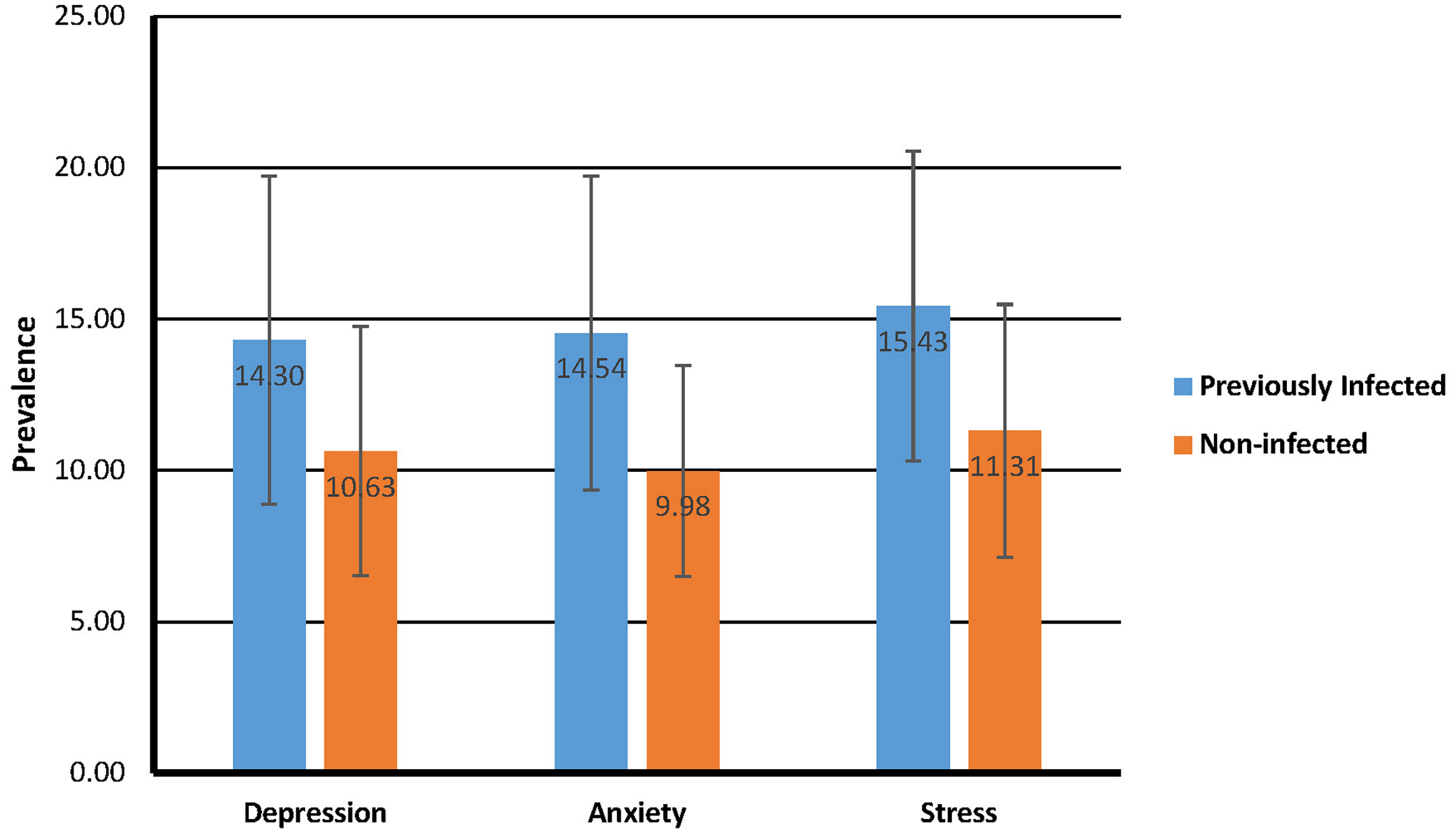
Infected vs non-infected comparison of DASS-21 mean scores.

An independent-samples *t*-test was also executed to measure the differences in depression, anxiety, and stress among those previously infected with SARS-CoV-2 and the non-infected participants (Figure 3). Significant differences existed between participants who had been infected with SARS-CoV-2 and those who had not been infected, for all three DASS-21 scales: Depression [*M* difference = 3.66, *SD* difference = 0.44, CI = 2.79–4.54, *t*(451.33) = 8.26, *p* = 0.000], Anxiety [*M* difference = 4.56, *SD* difference = 0.41, CI = 3.76–5.36, *t*(430.40) = 11.25, *p* = 0.000], and Stress [*M* difference = 4.12, *SD* difference = 0.43, CI = 3.27–4.97, *t*(459.58) = 9.57, *p* = 0.000]. Cohen’s *d* indicated that increases were large (Depression = 0.76; Anxiety = 1.02, Stress = 0.88). The test’s sensitivity was, therefore, realistic at 95% power.

## Discussion

Prior to the outbreak of the pandemic, several studies reported on employee mental health (Harvey et al., 2017; Greenwood et al., 2019; Kyron et al., 2019). The COVID-19 pandemic has seemingly exasperated the prevalence of declining mental health among employees. The psychological burden the pandemic placed on infected and uninfected employees in various industries is evident from the vast amount of research on the effect of the pandemic on psychological health and mental well-being. A title search of the terms ‘COVID-19 or coronavirus or 2019-ncov or sars-cov-2 or cov-19’ and ‘psychological well-being or mental health or psychological well-being’ in the EBSCOhost search engine generates more than 270 peer-reviewed academic articles. What is evident from the considerable COVID-19 research done in the psychological sphere is that, regardless of whether a person has been infected with SARS-CoV-2, the COVID-19 pandemic has significantly influenced psychological health and well-being.

The current study investigated the prevalence of burnout pre-and post-SARS-CoV-2 infection and determined whether differences in psychological health and well-being existed between SARS-CoV-2 infected and non-infected individuals. The difference in the level of burnout before and after having been infected with SARS-CoV-2 was statistically significant for Cognitive Weariness, Fatigue, and Emotional Exhaustion/Withdrawal. Like the findings of Hwang et al. (2021), Emotional Exhaustion/Withdrawal was the most prevalent, both pre-SARS-CoV-2and post-SARS-CoV-2 infection. The COVID-19 pandemic disturbed many people’s way of life, and if they were already experiencing burnout-related symptoms, the pandemic may have aggravated those symptoms. The uncertainty and fear surrounding the possibility of being infected with SARS-CoV-2 and the physical and emotional challenges that a person faces during actual infection seem to significantly increase emotional and interpersonal exhaustion and to increase detachment from work. Furthermore, participants’ levels of Cognitive Weariness, compared to Fatigue and Emotional Exhaustion/Withdrawal, increased the most from before to after SARS-CoV-2 infection. The outcomes of systematic literature reviews by Ceban et al. (2022) and Daroische et al. (2021) found cognitive impairment to be one of the most reported symptoms of the post-COVID-19 syndrome.

Participants who have been infected with SARS-CoV-2 reported significantly higher levels of burnout, anxiety, depression, and stress than those who have not been infected. While Emotional Exhaustion and Withdrawal were the most prevalent among both previously infected and non-infected groups, the most significant difference was the level of fatigue experienced. Brusaferri et al. (2022) found that the chronic stress of COVID-19 lockdowns may have caused neuroimmune activation in infected and non-infected individuals. Neuroinflammation can trigger symptoms such as mood alterations, mental and physical fatigue, discognition or “brain fog,” depression, and social withdrawal (Li et al., 2017; Brusaferri et al., 2022). Generally, burnout is caused by prolonged exposure to work-related stress (Koutsimani et al., 2019; Schaufeli, 2021). Interestingly, patients who suffered from long-COVID-19 were more likely to experience burnout (Selvaskandan et al., 2022). Coleman (2022) stated that long-COVID-19 can present similar symptoms to burnout and requires a different intervention approach. COVID-19 presented an unprecedented challenge to individuals, which exhausted the availability of their psychological, social and physical resources. Since well-being relates to the availability of psychological, social and physical resources an individual needs during a particular challenge, it makes sense that patients whose resources were depleted could show symptoms of burnout (Dodge et al., 2012).

In terms of anxiety, depression and stress, in both groups stress levels rose. However, a more significant increase took place in levels of anxiety. In their systematic literature review, Nagarajan et al. (2022) confirmed consensus across studies that people who survived severe SARS-CoV-2 infection were at a high risk of developing PTSD. A few studies also found that anxiety was significantly higher in participants who experienced a SARS-CoV-2 infection (Risal et al., 2020; Zhu et al., 2020; Broche-Pérez et al., 2021; Mohammadian Khonsari et al., 2021). During the pandemic, within the various stages of lockdown, people with medical conditions who had SARS-CoV-2 may have struggled to get to their medical appointments during or after infection (Broche-Pérez et al., 2021). According to Mohammadian Khonsari et al. (2021), people infected or previously infected with SARS-CoV-2 are more anxious about dying, infecting family or friends, being quarantined, and being re-infected with SARS-CoV-2.

### Theoretical implications

The biopsychosocial model was used to guide the current study in understanding the interaction between the COVID-19 pandemic, the SARS-CoV-2 infection, and psychological health and well-being. Within the principles of the biopsychosocial model, a previously infected person had to firstly deal with SARS-CoV-2, a biological event, and its physical consequences. Kevadiya et al. (2021) summarised the clinical presentation of SARS-CoV-2 infection and noted it could include “fever, sore throat, cough, chest and muscle pain, dyspnoea, confusion, anosmia, ageusia and headache. These can progress to life-threatening respiratory insufficiency, also affecting the heart, kidney, liver and nervous systems” (p: 593). Second, a previously infected person had to manage the psychological consequences of SARS-CoV-2 infection, which include increased levels of anxiety, stress, depression, burnout, trauma, and post-traumatic stress. A scoping review by Shanbehzadeh et al. (2021) that showed various physical and mental health problems such as anxiety, arthralgia, declines in daily functioning and activities, depression, fatigue, pain, post-traumatic stress disorder, and reduced physical capacity were present up to 3 months after SARS-CoV-2 infection. Lastly, a previously infected person had to endure social challenges such as isolation and quarantine due to their infection. Within the current study, the participants who have been infected with SARS-CoV-2 during the first 2 years of the pandemic were mandated by South African law to quarantine and stay in isolation for at least seven to 10 days.^3^ While a person who had not yet been infected could experience COVID-19-related psychological and social difficulties, given the additional biopsychosocial burden that an infected person experiences, it is understandable that there is a significant difference between the psychological health and well-being of these two groups.

### Practical implications and recommendations

Norris et al. (2002) found that mental health problems generally peaked a year after a disaster, followed by an improvement. Therefore, organisations must remain vigilant and implement psychological interventions to support distressed employees. The timely identification and precise diagnosis of psychological distress can aid in developing targeted psychological interventions for individuals exposed to a critical incident, such as the pandemic (Zhang et al., 2020). Mohammadian Khonsari et al. (2021) suggested that governments can help reduce the psychological burden of the COVID-19 pandemic and a SARS-CoV-2 infection by creating targeted interventions, distributing correct information about the psychological health and well-being effects of such an infection on various groups, and making a uniform COVID-19 mental health counselling service available to the public. Holmes et al. (2020) and Xiang et al. (2020) pointed out the importance of proactive steps to address the effect of COVID-19, which would likely require collaboration across disciplines, involving social workers, medical professionals and psychologists. Several studies point out that psychological counselling using electronic devices and applications (for example, smartphones and WeChat) and regular screening for depression, anxiety, and suicidal tendencies should be performed for COVID-19 patients, as well as health workers. An agile organisation that considers employee health, safety, and well-being, especially those infected with SARS-CoV-2, can survive and thrive in the uncertain times of the pandemic.

### Limitations and recommendations

The methodological limitations of this study include the cross-sectional and, more specifically, the retrospective nature of the study. The study used a retrospective approach to collect the pre-and post-SARS-CoV-2 burnout data. While retrospective studies have proved to be valid, reliable and useful, the descriptive and observational features of the research design limit the extent to which causal relationships between variables can be established (Talari and Goyal, 2020). Furthermore, recall bias, a type of systematic error, is introduced when asking participants to recall certain information and therefore relying on imperfect human memory to report certain events (Talari and Goyal, 2020) and as such the results should not be over-generalised to the whole population. It is suggested that future studies employ a longitudinal approach to see how the psychological health and well-being variables change over time. A procedural limitation of the current study is the use of a crowdsourcing platform to collect the data. According to Rice et al. (2017), disadvantages of using online populations include unrepresentative samples, financial motivation and potential fraud issues, limited length of study, and research can usually only measure attitudes and perceptions and not behavioural data. Alternatives to collecting data include using social media, personal networks, adverts, word of mouth, and emails. Another technical limitation is the lack of access to participants’ mental health history, which could bring a broader perspective or clearer context to the results. It is also important to research why some employees’ well-being improved during the pandemic, as a means for organisations to identify ways to support employees in the future (Campbell and Gavett, 2021).

## Conclusion

While there has been evident psychological well-being and mental health decline during the pandemic in general (Qiu et al., 2020; Restauri and Sheridan, 2020; Yıldırım and Solmaz, 2020; Campbell and Gavett, 2021; Mind Share Partners’ Mental Health at Work, 2021), being infected with SARS-CoV-2 significantly decreases a person’s psychological well-being and mental health.

## Data availability statement

The raw data supporting the conclusions of this article will be made available by the authors, without undue reservation.

## Ethics statement

The studies involving human participants were reviewed and approved by Department of Industrial Psychology and People Management, Research Ethics Committee, University of Johannesburg. The patients/participants provided their written informed consent to participate in this study.

## Author contributions

The author confirms being the sole contributor of this work and has approved it for publication.

## Conflict of interest

The author declares that the research was conducted in the absence of any commercial or financial relationships that could be construed as a potential conflict of interest.

## Publisher’s note

All claims expressed in this article are solely those of the authors and do not necessarily represent those of their affiliated organizations, or those of the publisher, the editors and the reviewers. Any product that may be evaluated in this article, or claim that may be made by its manufacturer, is not guaranteed or endorsed by the publisher.

## Footnotes

^1^For the purposes of this study, COVID-19 refers to the pandemic in general, while SARS-CoV-2 refers to being infected with the actual SARS-CoV-2 virus.

^2^ https://www.prolific.co/

^3^ https://www.gov.za/Coronavirus
